# Damaging Effects of Multi-walled Carbon Nanotubes on Pregnant Mice with Different Pregnancy Times

**DOI:** 10.1038/srep04352

**Published:** 2014-03-12

**Authors:** Wei Qi, Juanjuan Bi, Xiaoyong Zhang, Jing Wang, Jianjun Wang, Peng Liu, Zhan Li, Wangsuo Wu

**Affiliations:** 1 Radiochemistry Laboratory, Lanzhou University, Lanzhou, Gansu, China, 730000; 2 Department of Chemistry and Key Laboratory of Bioorganic Phosphorus Chemistry and Chemical Biology (Ministry of Education), Tsinghua University, Beijing, China, 100084; 3 Key Laboratory of Chemistry of Northwestern Plant Resources, Key Laboratory for Natural Medicine of Gansu Province, Institute of Chemical Physics, Chinese Academy of Sciences, Lanzhou, Gansu, China, 730000; 4 State Key Laboratory of Applied Organic Chemistry, Lanzhou University, Lanzhou, Gansu, China, 730000

## Abstract

The mechanism by which nanoparticles cross the placental barrier was studied by using isotopic tracers. The abortion rates and other related data were counted and analysed in pregnant mice with different pregnancy times. Results showed that oxidised multi-walled carbon nanotubes (oMWCNTs) crossed the placental barrier and entered the foetus body. The abortion rates in the pregnant mice depended on pregnancy times. The abortion rates in the first-time, second-time and fourth-time pregnant mice were 70%, 40% and 50%, respectively. The maternal body weight gain was inhibited until gestational ages of 13, 10 and 11 d. oMWCNTs decreased the serum progesterone level and increased the serum oestradiol level in a dose- and time-dependent manner. However, this effect decreased with gestational age. The histology and vascular endothelial growth factor/reactive oxygen species content in the placenta showed that oMWCNTs narrowed the blood vessel and decreased the number of blood vessels in the placenta.

Carbon nanomaterials, including carbon nanotubes (CNTs), graphene, nanodiamonds and fullerene, are widely employed in electrochemistry^1^,^2^,^3^,^4^,^5^, genetic engineering^6^,^7^, drug delivery^8^,^9^ and clinical diagnosis^10^,^11^. Evaluating the biological safety of carbon nanomaterials is important for their large-scale applications. Carbon nanomaterials induce apoptosis and DNA damage *in vitro*^12^,^13^. Their *in vivo* effects include inflammation and epithelioid granuloma formation in the lungs^14^,^15^, increased aortic plaque levels and atherosclerotic lesions in the brachiocephalic artery and mesothelioma formation in the abdominal cavity^16^,^17^.

A pregnant body or foetus is more sensitive to environmental toxins than adults. Many scholars have investigated the reproductive toxicity of nanomaterials^18^,^19^,^20^,^21^. Bai *et al.*^22^ found that repeated administrations of CNTs cause reversible testicular damage without affecting fertility in male mice. This finding suggests that the damaging effects of CNTs are lighter on the male reproductive system than on the female reproductive system. Consequently, researchers have focused on the effects of CNTs on the female reproductive system. However, CNTs must be capable of crossing the placental barrier to damage the placenta and foetus. Nanoparticles can cross the placental barrier from the maternal body into the offspring, influence the reproductive system, complicate pregnancy and cause nervous development problems in male foetuses^5^,^23^,^24^,^25^,^26^. These reports also indicated that the capability of nanoparticles to cross the placental barrier depends on their type, size and surface modification. Yamashita *et al.*^5^ detected silica and titanium dioxide nanoparticles in the placenta, foetal liver and foetal brain, suggesting that these nanoparticles can cross the placental barrier. Wick *et al.*^25^ employed the *ex vivo* human placental perfusion model to investigate whether or not polystyrene beads with diameters of 50, 80, 240 and 500 nm can cross the placental barrier or to determine whether or not this process is size dependent. They found that polystyrene particles with diameters of up to 240 nm can cross the placental barrier without affecting the viability of the placental explant. However, the toxic and damaging effects of nanoparticles on the mother and offspring remain unclear, respectively. Lim *et al.*^26^ evaluated the level of maternal toxicity in pregnant rats exposed to multi-walled carbon nanotubes (MWCNTs). They found that 14 d of repeated oral dosing of MWCNTs during pregnancy induce minimal maternal toxicity at 1000 mg/kg/d and that the no-observed-adverse-effect level of MWCNTs is 2000 mg/kg/d for dams and 1000 mg/kg/d for embryonic development. Yang *et al*.^21^ found that foetal exposure to nanoparticles in murine pregnancy is influenced by both embryonic/placental maturation stage and nanoparticle surface composition; however, they detected no signs of toxicity. Yamashita *et al.*^5^ found that silica and titanium dioxide nanoparticles with diameters of 70 and 35 nm can complicate pregnancy when injected intravenously into pregnant mice. They observed that mice treated with these nanoparticles have smaller uteri and smaller foetuses than untreated controls; they also discovered that fullerene molecules and large (300 nm and 1,000 nm) silica particles cannot induce these complications. Fujitani *et al*. indicated that various types of malformation were observed in all MWCNT-treated groups given intraperitoneally to pregnant ICR mice, but not observed such malformation until the MWCNT dose high to 4 or 5 mg/kg body weight by given intratracheally^27^. Clearly, nanoparticles can cross the placental barrier; however, the mechanism behind this process remains unclear. In addition, whether or not nanoparticles can damage the placental and foetal organs has yet to be explored. Some results showed that nanoparticles can cause significant maternal toxicity and foetal malformation^5^,^27^, whereas some reported otherwise^21^,^26^. To date, detailed statistical data about abortion rates induced by nanoparticles are lacking. Such data are important to assess the degree to which nanoparticles damage pregnancy. In addition, the relationship among abortion, placental malfunction and foetal malformation has yet to be established. Hence, the mechanism underlying foetal malformation and maternal toxicity remains unknown. In the present study, abortion was found related to the pregnancy times of mice, and recently, Albert *et al*.^28^ also found that the first-borns might be at a greater risk of metabolic and cardiovascular disease than second-borns, and they thought that those were attributed to the difference in placental blood flow and the structural changes occur to uterine spiral arteries to facilitate placentation during pregnancy. Thus, it was also included in the scope of the study.

In this work, we studied the mechanism by which CNTs cross the placental barrier by using radiotracers and determined the abortion rates caused by oxidised MWCNTs (oMWCNTs) in mice with different pregnancy times. We also investigated the maternal and foetal toxicity of oMWCNTs. The results of this study can help doctors alleviate the negative effects of nanomaterials and warn women who work or live in environments with heavy nanoparticles pollution to take safety measures during pregnancy.

## Results

### Biodistribution of ^99m^Tc-oMWCNTs in maternal tissue and foetus

**Figure 1:**
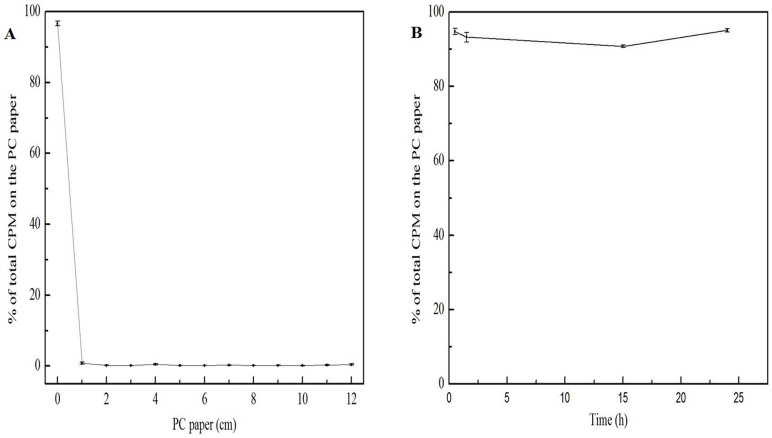
the determination radiolabling yields(A) and stability(B) of ^99m^Tc-oMWCNTs(by paper chromatography, and solvent was normal saline). (n = 4, all data represent means + s.e.m).

The radiolabelling yields and stability of ^99m^Tc-oMWCNTs were determined by paper chromatography, with normal saline as the solvent (Fig. 1). The radiolabelling yields were over 90%, and the radiolabelling compounds were very stable within 24 h *in vitro*. The lung, liver and placental tissues of the pregnant body and foetus were digested and then subjected to transmission electron microscopy (TEM) and Raman spectroscopy to further prove the reliability of the radiolabelling compounds. The oMWCNT structure is shown in Fig. S5.

**Figure 2:**
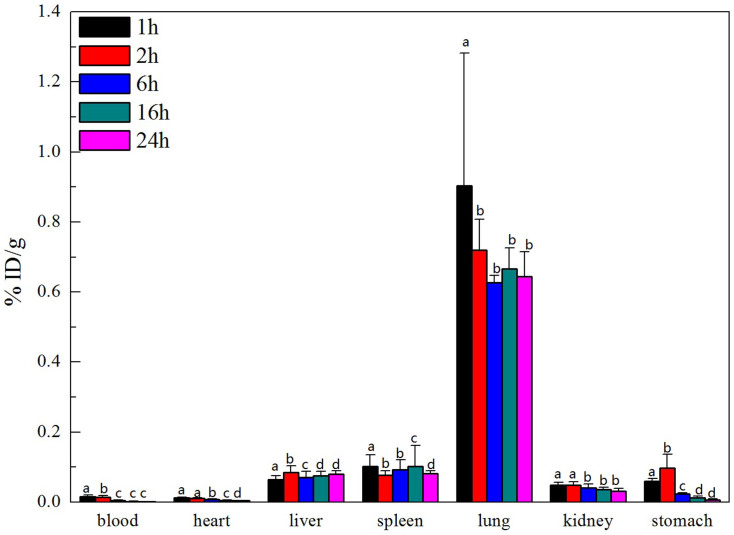
the biodistribution of oMWCNTs in pregnant mice at 1, 2, 6, 16 and 24 h post *i.v.* ^99m^Tc-oMWCNTs (GD = 18). (GD: gestational day, n = 5, all data represent means + s.e.m; the different alphabets is *p < 0.05 compared with each other in different time in the same organization by ANOVA).

**Figure 3:**
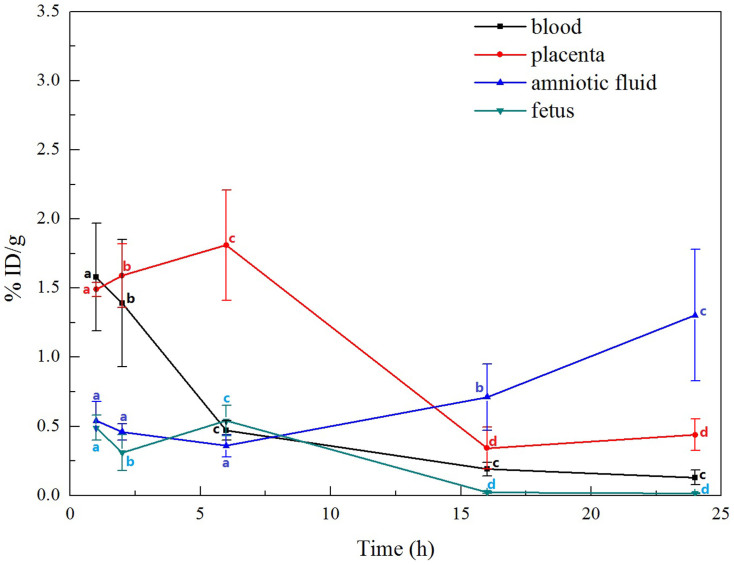
the clearance rates of oMWCNTs in amniotic, placenta, foetus and maternal blood at 1, 2, 6, 16 and 24 h post *i.v.*^99m^Tc-oMWCNTs. (n = 5–8, all data represent means + s.e.m; the different alphabets is *p < 0.05 compared with each other in different time in the same organization by ANOVA).

**Figure 4:**
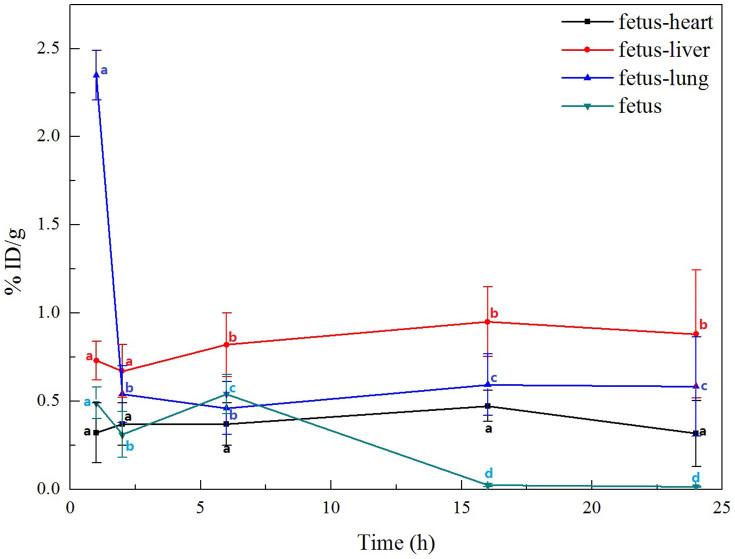
the clearance rates of oMWCNTs in heart, liver and lung of foetus and foetus at 1, 2, 6, 16 and 24 h post *i.v.* ^99m^Tc-oMWCNTs. (n = 8–10, all data represent means + s.e.m; the different alphabets is *p < 0.05 compared with each other in different time in the same organization by ANOVA or K-W test for foetus-heart).

The distribution of ^99m^Tc-oMWCNTs in the maternal and foetal bodies is shown in Figs. 2 to 4 (20 mg/kg.bw/mouse). In the maternal body, ^99m^Tc-oMWCNTs were principally distributed in the lungs (up to 90% ID/g), followed by the liver, spleen and kidney (Fig. 2). At 24 h after administration, the accumulation of ^99m^Tc-oMWCNTs decreased to 70% ID/g in the lungs, reduced to the minimum in the spleen and kidney, and slightly increased in the liver. The distribution of ^99m^Tc-oMWCNTs in the foetal body at 24 h after administration is shown in Fig. 3. The accumulation of ^99m^Tc-oMWCNTs was high in the placenta at 1 h after injection but low in the amniotic fluid and foetus. The accumulation of ^99m^Tc-oMWCNTs peaked in the placenta and foetus at 6 h after injection and then dramatically decreased at 6 h to 16 h after injection. The accumulation of ^99m^Tc-oMWCNTs in the amniotic fluid gradually increased in this process. These results indicate that oMWCNTs can pass through the maternal body into the foetus *in vivo*.

Foetal liver is the direct receptor of maternal blood^29^. Organs with the mononuclear phagocytic system easily absorb and accumulate ^99m^Tc-oMWCNTs^30^. Therefore, we hypothesised that foetal liver easily absorbs ^99m^Tc-oMWCNTs from maternal blood. The foetal heart, liver and lungs were harvested to evaluate their radioactivity and to further prove whether or not ^99m^Tc-oMWCNTs can enter the foetal body. Fig. 4 shows that ^99m^Tc-oMWCNTs highly accumulated in the foetal lungs at 1 h, dramatically decreased and peaked at 2 h, and then stabilized until 24 h. The accumulation of ^99m^Tc-oMWCNTs in the heart and liver slightly increased and then stabilized.

### Abortion and fertility rates of pregnant mice with different pregnancy times after injection with oMWCNTs

**Figure 5:**
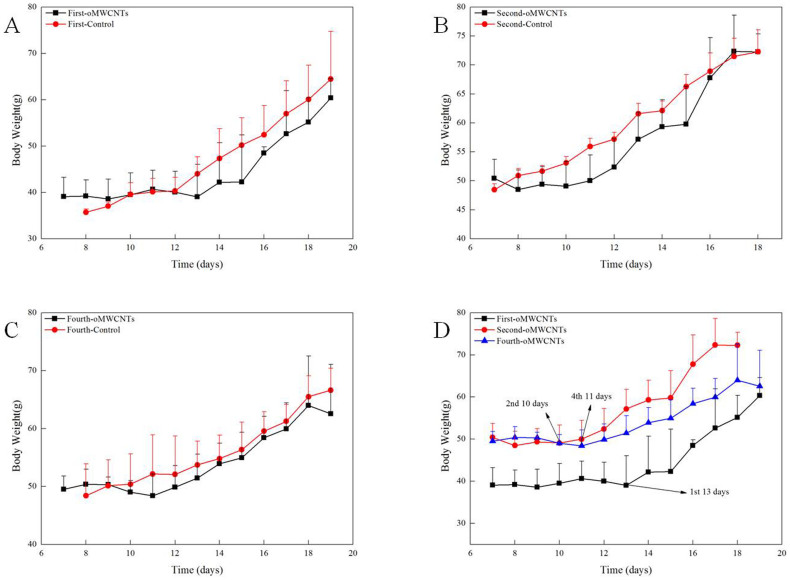
changes of maternal body weight in pregnancy (exposure groups with oMWCNTs 20 mg/kg.bw, body weights were evaluated daily. (A), first pregnant mice with exposure (first-oMWCNTs) and control groups (first-control). (B), second pregnant mice with exposure (second-oMWCNTs) and control groups (second-control). (C), fourth pregnant mice with exposure (fourth-oMWCNTs) and control groups (fourth-control). (D), the combination picture of first, second, and fourth pregnant mice during pregnancy with exposure to oMWCNTs (n = 10, all data represent means + s.e.m).

**Figure 6:**
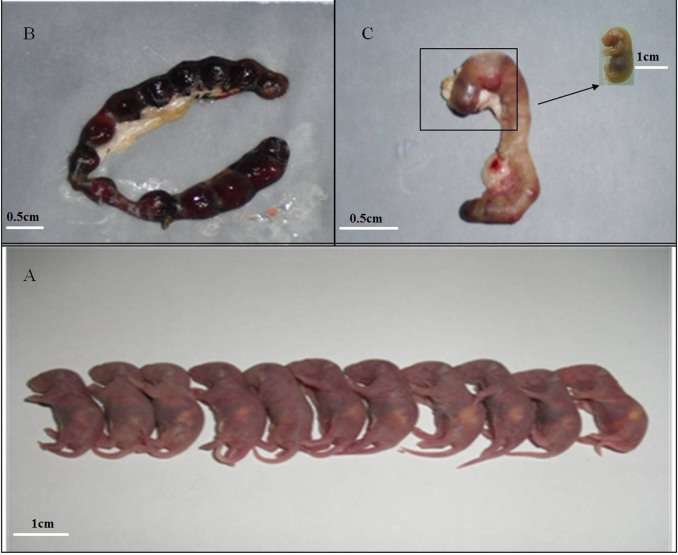
the effect of oMWCNTs (20 mg/kg.bw) on foetal development after successive exposure to pregnant mice. (A), foetus of the normal parturition. (B), the foetus of abortion for the first pregnant mice. (C), the foetus of abortion for the second and fourth pregnant mice.

The effect of oMWCNTs on pregnant mice with different pregnancy times was studied (oMWCNTs: 20 mg/kg.bw through intravenous injection) by determining abortion and fertility rates. The experimental results are shown in Fig. 5. Compared with the control group, the exposure groups had poor embryo development and had inhibited maternal body weight gain during the first few days after exposure. Then, the maternal body weight suddenly increased at the gestational ages of 13, 10 and 11 d, and continued to abortion or production, but was always lower than that in the control group. These results are consistent with the results shown in Fig. 5. The foetal weights of the exposure group was also smaller than that of the control group during pregnancy (Fig. 6). As shown in Fig. 6b, miscarriage occurred in the early stage of embryo development in the first-time pregnant mice. As shown in Fig. 6c, the foetuses in the uterus died after abortion in the second-time or fourth-time pregnant mice. However, the development of the dead foetus was better in the second-time or fourth-time pregnant mice than that in the first-time pregnant mice and similar to that in the normal foetus from the parturition in the control groups (Fig. 6a). As shown in Table 1, the abortion rates in the first-time, second-time and fourth-time pregnant mice post-intravenously injected with oMWCNTs (20 mg/kg.bw) were 70%, 40% and 50%, respectively, whereas those in the control groups were 10%, 0% and 30%, respectively. The average weight changes in the pregnant mice before and after production are also shown in Table 1.

### Effect of oMWCNTs on progestational hormone level in maternal serum

#### Effect of exposure doses on progesterone and oestradiol levels in maternal serum

**Figure 7:**
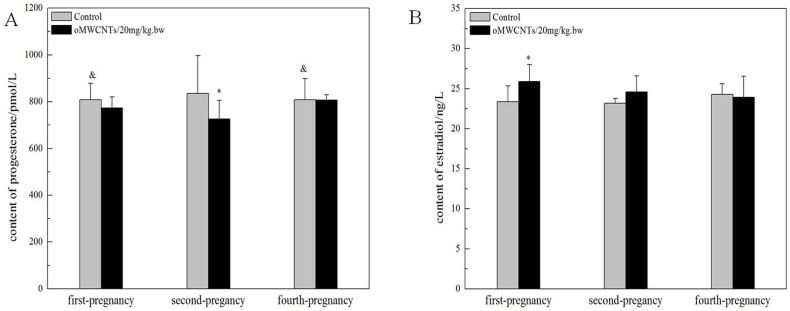
the effects of oMWCNTs (20 mg/kg.bw) on progesterone (A)/or estradiol (B) of pregnant mice with different pregnant times. *p < 0.05 compared with the control groups.^&^*p* < 0.05 compared with the control group of second-pregnancy(n = 6–8, all data represent means + s.e.m, all data were statistical analysis by ANOVA).

**Figure 8:**
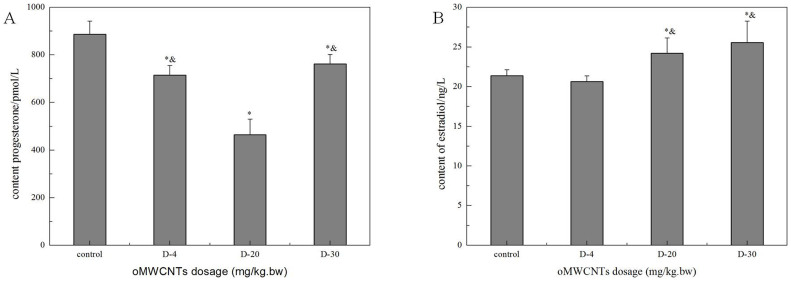
the effect of oMWCNTs dosage on progesterone (A)/or estradiol (B) for GD 14 days pregnant mice. 4 mg/kg.bw: (D-4); 20 mg/kg.bw: (D-20); 30 mg/kg.bw: (D-30). (A), *p < 0.05 compared with the control groups; ^&^*p* < 0.05 compared with the D-20 group. (B), *p < 0.05 compared with the control groups; ^&^*p* < 0.05 compared with the D-4 group (n = 4–5, all data represent means + s.e.m, all data were statistical analysis by ANOVA).

The effect of oMWCNTs on pregnancy was studied by exposing pregnant mice to oMWCNTs (4, 20 and 30 mg/kg.bw) at the gestational age of 14 d. Fig. 7 shows the progesterone and oestradiol levels in maternal serum after abortion. The serum progesterone level in the first-time pregnant mice from the exposure group was lower than that in the mice from the control group, whereas the serum oestradiol level in the first-time pregnant mice from the exposure group was significantly higher than that in the mice from the control group. Compared with the control groups, the exposure groups showed reduced serum progesterone level and increased serum oestradiol level after oMWCNT injection in a dose-dependent manner (Fig. 8). The serum progesterone level decreased to the minimum after injection with 20 mg/kg.bw oMWCNTs. Meanwhile, similar serum progesterone levels were observed in the mice injected with 30 and 4 mg/kg.bw oMWCNTs (Fig. 8a).

#### Effect of oMWCNTs on progesterone and oestradiol levels in maternal serum at different gestational ages

**Figure 9:**
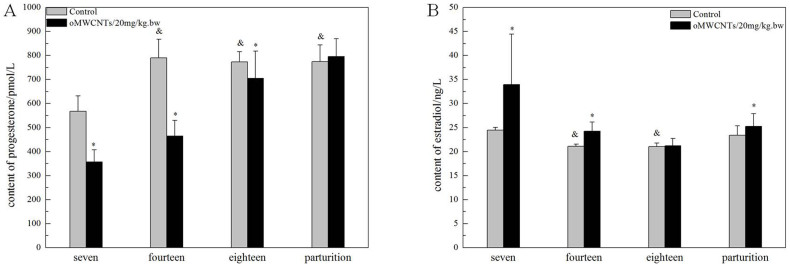
the effect of oMWCNTs (20 mg/kg.bw) on the progesterone (A)/or estradiol (B) in the mice at GD 7 days, 14days, and 18 days. (A), *p < 0.05 compared with the control groups; ^&^*p* < 0.05, compared with the control group of GD seven days. (B), *p < 0.05 compared with the control groups, ^&^*p* < 0.05 compared with the control group of GD seven days and the control group of parturition (n = 5–6, all data represent means + s.e.m, all data were statistical analysis by ANOVA).

The secretion of progesterone and oestradiol highly depends on the different stages of pregnancy in mammals^31^. The pregnant mice were exposed to 20 mg/kg.bw oMWCNTs at the gestational ages of 7, 14 and 18 d. Then, the levels of progesterone and oestradiol in the maternal serum were measured (Fig. 9). As shown in Fig. 9a, the serum progesterone level was lower in the exposure groups than in the control groups at the gestational ages of 7, 14 and 18 d (*p < 0.05 vs. control groups). This result indicated that oMWCNTs inhibited the secretion of progesterone after entering the placental system (Fig. 9a). This effect was gradually weakened with gestational age. As shown in Fig. 9b, the serum estradiol level in the exposure groups was higher than that in the control groups at the gestational ages of 7 d or 14 d. The oestradiol level in the exposure groups was close to that in the control groups at the gestational age of 18 d.

#### Effects of exposure time on progesterone and oestradiol levels in maternal serum

**Figure 10:**
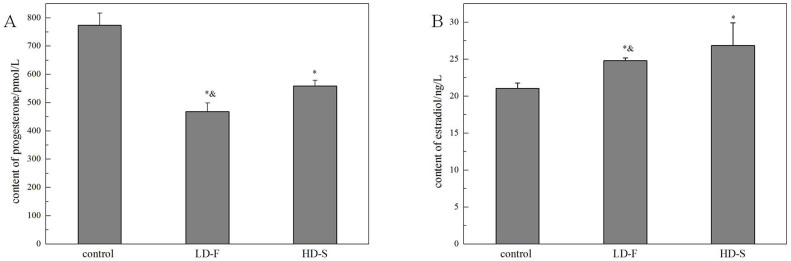
the effect of exposure time on the progesterone (A)/estradiol (B) in the mice at GD 18 days post injection with oMWCNTs. (A–B),*p < 0.05 compared with the control groups; ^&^*p* < 0.05, compared with the HD-S. LD-F is exposure group with low dosage (4 mg/kg.bw/per day) five times for 5 days. HD-S is exposure group with heavy dosage (20 mg/kg.bw) for 1 day (n = 5, all data represent means + s.e.m, all data were statistical analysis by ANOVA).

As shown in Fig. 10a, the serum progesterone level was significantly lower in the exposure groups than in the control groups (*p < 0.05). Multiple exposure to a low dose (4 mg/kg.bw/d, five times in 5 d) further increased the serum progesterone level than single heavy exposure (20 mg/kg.bw/d, once in 5 d, *p < 0.05). As shown in Figs. 10a and 10b, the serum oestrogen level was significantly higher in the exposure groups than in the control groups (*p < 0.05). The serum oestrogen level in the exposure groups injected once with a heavy dose was higher than that in the exposure groups injected multiple times with a low dose (*p < 0.05).

### Damage of oMWCNTs on foetal organs and placenta

#### Level of reactive oxygen species (ROS) and vascular endothelial growth factor (VEGF) in the placenta after injection with oMWCNTs

**Figure 11:**
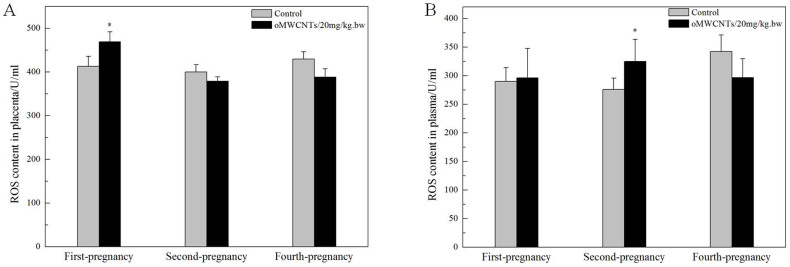
the content of ROS in placenta (A)/plasma (B) at about GD 14 days. (A–B),*p < 0.05 compared with the control groups (n = 5–6, all data represent means + s.e.m, all data were statistical analysis by ANOVA).

**Figure 12:**
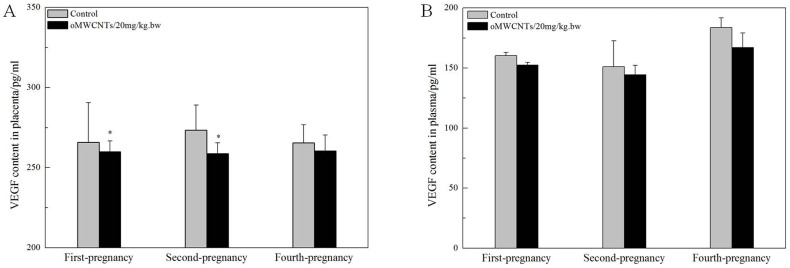
the content of VEGF in placenta (A)/plasma (B) at about GD 14 days. (A–B)*p < 0.05 compared with the control groups (n = 5–6, all data represent means + s.e.m, all data were statistical analysis by ANOVA).

**Figure 13:**
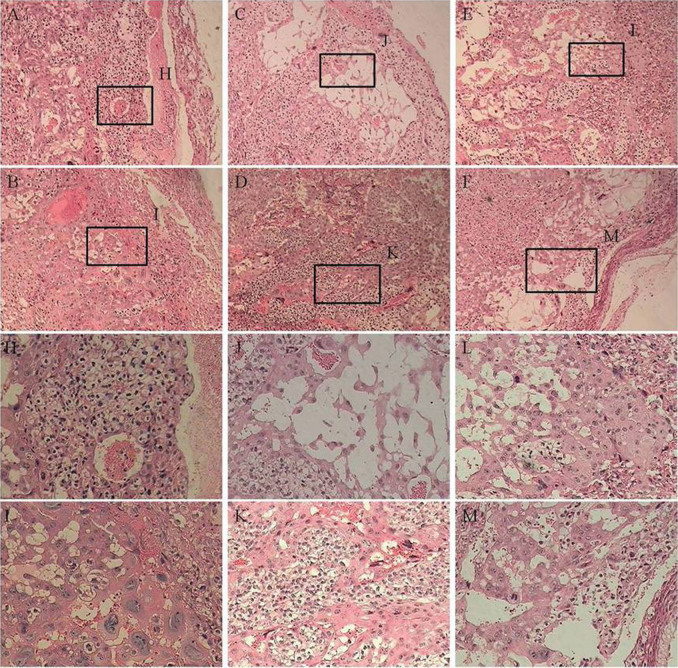
the histology of placental. (×4) (A–F) and (×10) (H–M), and (A), (C), (E) is control groups of first, second and fourth pregnancy, respectively. (B), (D), (F) is exposure groups of first, second and fourth pregnancy, respectively. (H–M) is the high power field of the designated area of (A–F), respectively.

**Figure 14:**
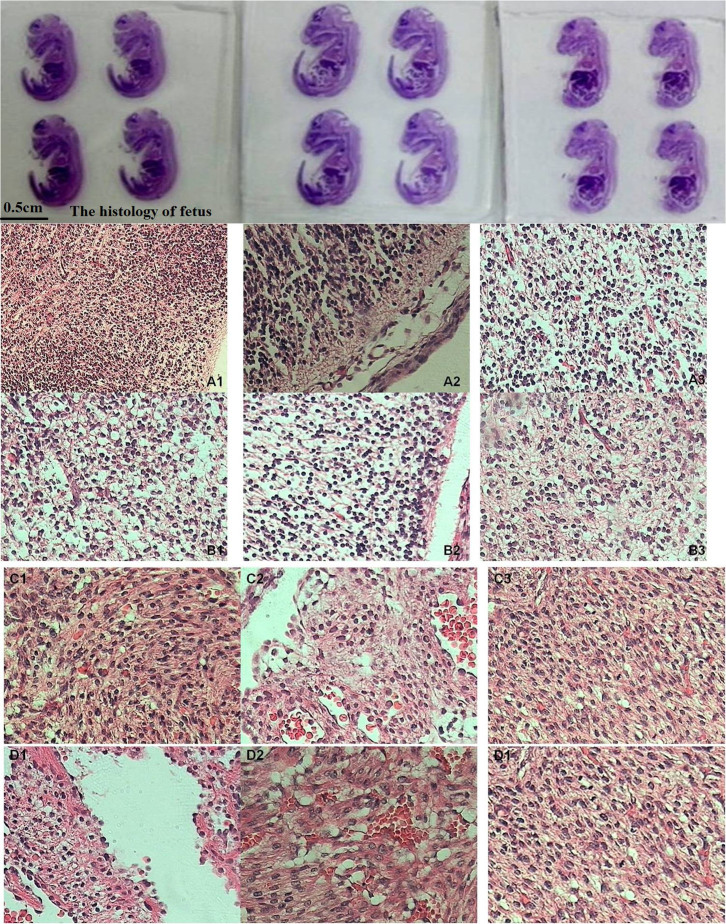
the histology of brain and heart of foetus (×40). (A1), (A2), (A3) is the brain tissues for foetus of normal groups of first, second and fourth pregnancy, respectively. (B1), (B2), (B3) is the brain tissues for foetus of exposure groups of first, second and fourth pregnancy, respectively. (C1), (C2), (C3) is the heart tissues for foetus of normal groups of first, second and fourth pregnancy, respectively. (D1), (D2), (D3) is the heart tissues for foetus of exposure groups of first, second and fourth pregnancy, respectively.

The damaging effects of oMWCNTs on the placenta and foetus were also examined, and the results are shown in Figs. 11 to 14. The placental content of ROS significantly decreased in the group exposed to 20 mg/kg.bw oMWCNTs compared with that in the control group for the first-time pregnant mice. However, no obvious differences were observed in placental tissues for the second-time and fourth-time pregnant mice (Fig. 11a). No obvious differences in the plasma ROS content were observed between the control and exposure groups. The placental content of VEGF was lower in the exposure groups than in the control groups, except for the fourth-time pregnant mice (Fig. 12).

#### Histological observation of placental tissues after injection with oMWCNTs

As shown in Fig. 13, the cell size increased and the number of blood vessels decreased in the placental tissue from the exposure groups compared with those from the control groups of the first-time pregnant mice. For the second-time and fourth-time pregnant mice, the placenta showed minimal lesion disease in cells and had abundant blood vessels in the control groups. However, in the exposure groups, the placenta showed tissue oedema and even some anucleate cells. The number of blood vessels also decreased and narrowed in the exposure groups. As shown in Figs. S7 to S9, the lungs and liver of the foetus were not affected after the pregnant mice were exposed to 20 mg/kg.bw oMWCNTs. As shown in Fig. 14, the heart and brain of the foetus were damaged after the pregnant mice were exposed to 20 mg/kg.bw oMWCNTs.

## Discussion

Radiotracers are usually used to investigate the fate and behaviour of nanomaterials because of their high sensitivity, credibility and resistance to interference^32^,^33^,^34^. In the present study, ^99m^Tc-labelled oMWCNTs with diameters of 20 nm to 30 nm (Fig. S1) were investigated for their behaviour and fate in pregnant mice. The oxidized CNTs contained many OH− and COOH− groups (Figs. S2–S3). Thus, the unfilled electron orbits of Tc(V) were filled immediately by electrons donated by four hydroxy groups from oMWCNTs. Stable chelate complex ^99m^Tc-oMWCNTs were formed with high labelling rate and long-term stability (Fig. 1). After single exposure to Na^99m^TcO_4_, most are cleared quickly by urine without obvious retention in the lungs and other organs^35^. However, in the present study, ^99m^Tc-oMWCNTs highly accumulated in the lungs, liver and other organs of the maternal or foetal body at 24 h after injection. These results suggest that the labelling compounds have tracer properties and can effectively respond to the behaviour of oMWCNTs *in vivo*.

After the intravenous injection of ^99m^Tc-oMWCNTs (20 mg/kg.bw), they loaded the venous blood from the right atrium into the left ventricle, the middle part of which included the pulmonary capillary bed. Most oMWCNTs were captured by the pulmonary capillary bed and highly accumulated in the lungs at 1 h^36^ (Fig. 2). Some ^99m^Tc-oMWCNTs in the lungs were eliminated and entered the circulatory system with time, whereas some were absorbed by macrophage organs (i.e., spleen, liver). As a result, high accumulation of ^99m^Tc-oMWCNTs was observed in the spleen and liver (Fig. 2). These results are consistent with several previous reports^5^,^16^,^30^. Some nanoparticles entered the placenta through the circulatory system, and the placental content of nanoparticles started to increase at 1 h to 6 h after injection. The placenta is the main organ for the exchange of materials between the maternal body and the foetus, and the amniotic membrane separates the materials between the maternal body and amniotic fluid. Some nanoparticles could cross the placental barrier into the foetus, whereas some could permeate the amniotic fluid *via* the exchange of materials in the amniotic membrane (Fig. 3). After entering the foetus, most ^99m^Tc-oMWCNTs were captured by the pulmonary capillary bed and accumulated in the foetal lung at 1 h after injection (Fig. 4). These nanoparticles were then rapidly removed into other foetal tissues through the circulatory system. The content of these nanoparticles increased in the liver and heart (Fig. 4). The liver contains many macrophage organs; thus, the distribution of nanoparticles in the liver is greater than that in the heart. After injection, some nanoparticles penetrated into the foetal body through the exchange of materials between the amniotic fluid and the foetus, inducing a downward tendency for the content in amniotic fluid (Fig. 4). At 6 h after injection, the placental content of the nanoparticles rapidly decreased because of the decrease in maternal supply (Fig. 3). The foetus excreted the nanoparticles through the alimentary canals into the amniotic fluid; as a result, the nanoparticle distribution gradually decreased in the foetus and continuously increased in the amniotic fluid until 24 h (Fig. 3). Possible transport routes for nanoparticles across the placenta include diffusion, vesicular transport, transmembranal transporter proteins and the transtrophoblastic channel system^25^. Diffusion and vesicular transport are only applicable for nanoparticles less than 120 nm in size, whereas the transtrophoblastic channel allows the transport of liquid and small molecules. oMWCNTs with diameters of 20 nm to 30 nm and lengths of 1 μm to 2 μm were utilised in the present study (Fig. S1). Placenta transport proteins were involved in the mechanism by which ^99m^Tc-oMWCNTs crossed the placental barrier. As shown in Fig. S6, the content of ^99m^Tc-oMWCNTs (ID/g%) in the foetus was significantly lower at the gestational age of 14 d (^99m^Tc-oMWCNTs 20 mg/kg.bw exposure) than at the gestational age of 18 d. This result suggests that the capability of ^99m^Tc-oMWCNTs to cross the placental barrier improves with time. Enders and Blankenship^29^ hypothesised that the placental barrier thickness decreases and the foetal capillary number increases in the late phase of pregnancy. These phenomena can possibly decrease the effect of the placental barrier and improve the placental transport ability of nanoparticles. As shown in Fig. S5, oMWCNTs accumulated in the lungs, liver, foetus, and placenta of the pregnant mice. These results suggest that CNTs are more dangerous for pregnancy at the late stage of gestation. Therefore, pregnancy at this stage must be monitored.

As shown in Figs. 5a to 5c, the maternal body weight of the control pregnancy groups increased starting from the gestational age of about 8 d. However, the first-time, second-time and fourth-time pregnant mice showed a delay in weight gain before the gestational age of 13, 10 or 11 d, respectively, after successive exposure to oMWCNTs (20 mg/kg.bw). The delay in weight gain may be due to delayed implantation or foetal development disorder^31^. Delayed implantation leads to delayed embryonic development and thus delayed weight gain. The pregnancy time could extend for over 21 d^37^. However, in the present study, the pregnancy time did not extend and reached a full 21 d. Hence, the delay in bodyweight gain is not due to delayed implantation. Normal placental development is required for embryonic growth, and placental dysfunction is associated with miscarriage and foetal growth restriction^38^,^39^. Thus, the delay in maternal body weight gain principally resulted from placental dysfunction after injection with oMWCNTs which caused placental damage. The observed placental damage would be discussed in detail later (Figs. 11 to 14). As shown in Fig. 5d, the longest delay in weight gain occurred in the first-time pregnant mice (13 d). This result suggests that the delay in body weight growth strongly depends on the pregnancy times of the mice. Moreover, the most severe damaging effects of oMWCNTs on pregnancy can be observed in the first-time pregnant mice.

In the exposure groups, the development of dead foetus after abortion (20 mg/kg.bw oMWCNTs) was better in the second-time or fourth-time pregnant mice (Fig. 6b) than in the first-time pregnant mice and was even close to the normal foetus (Fig. 6a). The foetuses of the first-time pregnant mice from the exposure group were very small and had poor development. This result suggests that abortion might occur in the first-time pregnant mice at the early phase of foetal development. A similar result was observed by Pietroiusti *et al.*^20^. Meanwhile, abortion occurred in the second-time or fourth-time pregnant mice at the late phase of foetal development. Thus, the foetal development in the second-time or fourth-time pregnant mice was better than that in the first-time pregnant mice after exposure to nanoparticles. These phenomena might be determined by the different pregnancy conditions for the first-time, second-time and fourth-time pregnant mice^28^. Moreover, the higher progesterone level in the second-time pregnant mice than the first-time or fourth-time pregnant mice (Fig. 7a) might confer resistance against the negative influence of oMWCNTs.

Table 1 shows the abortion rates and the average weight change before and after production after successive exposure to oMWCNTs (20 mg/kg.bw). In the present study, we employed the average body weight change before and after production to show the number of abnormal foetuses absorbed *in vivo*. The average weight change before and after production is mainly due to the negative effect of oMWCNTs on fecundity. Small weight change before and after production suggests that more embryos or foetuses have been absorbed in the uterus before production or abortion. Specifically, oMWCNTs cause severe damage on pregnancy. As shown in Table 1, the average weight change of the first-time pregnant mice from the exposure groups (8.57 ± 8.95 g) before and after production was significantly lower than that of the mice from the control groups (20.73 ± 9.00 g). However, the other exposure groups had no significant differences when compared with their corresponding control groups. This result indicated that more foetuses or embryos were absorbed *in vivo*. In addition, the high abortion rate of 70% occurred correspondingly in the early part of pregnancy of the first-time pregnant mice from the exposure groups (Table 1 and Fig. 6b). The damage of oMWCNTs on the first-time pregnant mice was more severe than on the other mice. It also delayed their bodyweight growth until 13 d (Fig. 5). The difference in average weight for the control group of the first-time pregnant mice (20.73 ± 9.00 g) was higher than that for the control group of the second- and fourth-time pregnant mice (15.83 ± 4.24 g and 12.72 ± 5.78 g, respectively). The abortion rate of the first-time pregnant mice (10%) was slightly higher than that of the second-time pregnant mice (0%) but significantly lower than that of the fourth-time pregnant mice (50%) from the control group. This condition were attributed to the difference in physiological functions^28^. Elderly women are prone to miscarriage during pregnancy^40^,^41^. The abortion rates reached 30% of the fourth-time pregnant mice from the control group (Table 1). However, the 50% abortion rate of the fourth-time pregnant mice from the exposure groups was not high when compared with the 30% abortion rate of the control group. This result suggests that an increase in the frequency of pregnancy can decrease their sensitivity to oMWCNT exposure during pregnancy, and which was accordance with the recent research results^28^, they mentioned that structural changes occurred to uterine spiral arteries to facilitate placentation, and the changes did not reverse in following parturition, suggesting a more favourable foetal environment for subsequent pregnancies. Given that the nanoparticles can affect the growth of foetuses and cause high abortion rates, we investigated the damage mechanism by determining biochemical indexes.

Progesterone and oestrogen have important functions in maintaining normal pregnancy and in initiating parturition with modulation myometrial contractility and excitability^42^,^43^,^44^. The serum progesterone and oestradiol levels were measured to demonstrate whether and how oMWCNTs affect the normal pregnancy of mice. oMWCNTs damaged the placental tissue (Fig. 13) and stimulated the immune cells in the placenta to generate immune responses by crossing the placental barrier and entering the placenta. The placenta then secreted large quantities of Hofbauer cells and anti-inflammatory hormone factors (such as glucocorticoid). The increase in glucocorticoids promoted the secretion of placental hormone releasing hormone, causing the decline in progesterone (Fig. 8)^45^,^46^,^47^. As the dose of oMWCNTs was increased, more nanoparticles entered the placenta, causing the decline in the content of secreted progesterone (Fig. 8). When the dose of oMWCNTs was increased to 30 mg/kg.bw, the nanoparticles agglomerated and accumulated in the maternal liver and lungs^30^, which further reduced the concentration of oMWCNTs in maternal blood and placenta (Fig. 8). Hence, the effects of the nanotubes were weakened. Some studies indicated that progesterone decreases the expression of the oestrogen receptor to lower oestrogen response and that oestrogen increases the expression of the progesterone receptor in uterine to improve progesterone response^48^,^49^,^50^. This system ensures that hormones could be secreted in a relatively wide range, thereby allowing the intrauterine environment to be in favour of the embryo's survival and implantation. As the gestational age was prolonged, the level of progesterone continuously increased before the gestational age of 14 d, but the oestradiol level initially decreased, stabilised and then rapidly increased because of cervical contraction and foetal production before delivery^44^. The progesterone level started to decline with parturition and placenta shedding, but this result was different from previous experiments^51^. The discrepancy in results can be attributed to different experimental conditions, such as animal species (Fig. 9a). Accordingly, oMWCNTs can affect the secretion of progestational hormones and the pregnancy of mice. They also negatively depend to some extent on the dosage and the growth of gestational age.

In an actual environment, many pregnant bodies often have to work and/or live in environments with light pollution of nanoparticles or other impurities for long periods. Therefore, determining whether or not the exposure time of oMWCNTs can affect normal pregnant processes is necessary (Figs. 10a and 10b). Some oMWCNTs in maternal blood circulation crossed the placental barrier and entered the placenta, thereby causing changes in placental function (Figs. 11 to 13). This phenomenon decreased the progesterone level and increased the oestrogen level of all exposure groups (Fig. 10). Low contents of nanoparticles were observed in the blood of mice exposed to high oMWCNT dose (20 mg/kg.bw/d)^36^ because the suspension of these nanotubes was easily agglomerated. As a result, a few oMWCNTs entered the placental tissue. Meanwhile, high contents of nanoparticles were detected in the blood of mice exposed to low oMWCNT dose (4 mg/kg.bw/d) because of the low agglomeration of these nanotubes and because of successive exposure. As a result, many oMWCNTs entered the placental tissue. The secretion of progesterone was more obviously inhibited in the mice with multiple exposure to a low dose than in the mice with a single exposure to a heavy dose (Fig. 10a). According to previous studies^52^, the level of oestrogen (e.g., oestradiol) gradually increases as the level of progesterone decreases in the blood. This result is consistent with the findings demonstrated in Fig. 10b. oMWCNTs entering the body could stimulate tissues to secrete many macrophages and immune factors, including steroid hormone^30^,^32^. The placenta is an incomplete endocrine organ but is a vital organ in oestrogen synthesis. The raw materials (e.g., steroid hormone) used to synthesise and secrete oestrogen originate from the maternal body^53^. Most oMWCNTs were retained in the maternal tissues because of agglomeration after the one-time exposure to a heavy dose. The maternal organs produced strong immune responses against oMWCNTs and stimulated further hormone secretion; these phenomena led to increased levels of steroid and protein hormones in the maternal blood. These materials were adopted easily to synthesise oestrogen in the placenta and to increase oestrogen level, thereby inhibiting progesterone secretion to some extent (Fig. 10b). ROS are important regulators of vascular function; high ROS levels can cause cell apoptosis or tissue necrosis. As shown in Fig. 11a, oMWCNTs clearly decreased the ROS content in the placenta of the first-time pregnant mice but not of the second-time and fourth-time pregnant mice (Fig. 11a). This result could be attributed to the pathological changes in the placenta for both the exposure and control groups of the second-time/fourth-time pregnant mice (Fig. 13). As shown in Fig. 11b, the ROS content in the plasma of the exposure groups had no obvious differences when compared with that in the plasma of the control groups, suggesting that oMWCNTs induced minor lesions of the maternal body (Fig. S6). VEGF can affect the number of blood vessels in the placenta. The placental content of VEGF in the exposure groups was lower than that in the control groups, except for the fourth-time pregnant mice. This result indicates that oMWCNTs can reduce the amount of placental blood vessels (Fig. 12), as confirmed from the histological pathology (Fig. 13). Vascular reduction in the placenta affects the supply of oxygen and nutrition for the foetus. As a result, foetal development was delayed; the weight growth of the maternal body was also delayed until the gestational ages of 13, 10 and 11 d for the first-time, second-time and fourth-time pregnant mice, respectively (Fig. 5). Foetal dysplasia causes histopathological changes in the organs, and the changes in the contents of ROS and VEGF in the blood were confirmed by Pietroiusti and Yamashita^5^,^20^. However, all previous works failed to clarify the causation among abortion, placental lesions and foetal malformation^5^,^20^,^21^,^26^,^27^,^29^. We performed histopathological observation of the maternal tissue, whole foetus and placenta (Figs. 13–14, S7–S9) to clarify the problem. As shown in Fig. 14, cell necrosis in the heart and brain of the foetus occurred after the pregnant mice were exposed to oMWCNTs. This result can be attributed to the fact that the heart and brain are tachyaerobic organs with high metabolic rates. In particular, the heart and brain of the foetus need more nutrients for development during pregnancy. However, oMWCNTs decreased the numbers of blood vessels in the placenta, causing foetal hypoxia and further decreasing the nutrient transport ability of the placenta. This condition also damaged the brain and heart tissues of the foetus. Long-term exposure of pregnant mice to oMWCNTs can cause foetal growth retardation (Figs. 5a–5c), embryonic death or even abortion (Table 1). As shown in Figs. S7–S9, the lung and liver tissues were normal after injection with oMWCNTs, which might have resulted from hypoplastic lung and liver of powerful detoxification function for the foetus. In short, the pathological changes, such as foetal death induced by brain and heart tissues, caused abortion and absorption.

The oMWCNTs administered to the mice at the gestational age of 14 d crossed the placental barrier and caused placental tissue damages, such as trophoderm cell swelling or apoptosis. The number of blood vessels in the placenta tissue decreased (Fig. 13). This phenomenon inhibited progesterone secretion so that the pregnancy cannot effectively reject the adverse factors caused by placental dysfunction^54^. This phenomenon also promoted oestradiol secretion and then stimulated the excitability of uterine contraction which was disadvantageous for pregnancy. Subsequently, pregnancy problems (e.g., restriction of foetal growth, absorption and abortion) occurred in the exposure groups (Figs. 5d and 56). Albert *et al.*^28^ also speculated that the influences of pregnancy times to foetus might be attributed to the differences in placental blood flow during pregnancy, and this results also confirmed their speculation.

In summary, nanoparticles can clearly complicate pregnancy. The level of nanoparticles should be controlled strictly in environments where pregnant women usually live or work. Aside from avoiding long-term exposure, pregnant women (especially first timers) in environments with heavy pollutionmust be subjected to regular pregnancy tests and must be injected with appropriate amounts of progesterone or other hormones to protect their pregnancy.

## Conclusion

After exposure to pregnant mice through intravenous injection, oMWCNTs could cross the placental barrier into the foetus and mainly accumulate in the foetal liver, lungs and heart. oMWCNTs decreased progesterone levels and increased oestradiol levels in the serum. The effects of oMWCNTs on pregnancy depended on the exposure dose but weakened with gestational age. The damaging effects of oMWCNTs were more serious on first-time pregnant mice than on multiple pregnancy mice. oMWCNTs damaged placental dysfunctions, thereby delaying foetal growth and further harming the foetal heart and brain. This condition could lead to abortion. These results suggest that CNTs elicit severe toxicity to pregnant women. Proper safety precautions must be taken to prevent damages caused by carbon nanoparticle pollution during pregnancy.

## Methods

### Preparation of oMWCNTs

MWCNTs prepared by chemical vaporisation deposition were commercially obtained from Shenzhen Nanotech Port Co. Ltd., Guangdong, China. According to the product specification, as-received MWCNTs were determined by TEM to be 1 μm to 2 μm in length and 10 nm to 30 nm in diameter. Purity was > 96% (wt.%), containing < 3% amorphous carbon and < 0.2% ash. The as-grown MWCNTs (called untreated MWCNTs) were added into the solution of 3 mol/L HNO_3_ to remove the hemispherical caps of the nanotubes. The mixture of 3 g MWCNTs and 400 mL 3 mol/L HNO_3_ was ultrasonically stirred for 24 h. The suspension was filtered, rinsed with deionised water until the pH of the suspension reached approximately 6, and then dried at 80°C. The oMWCNTs were then characterised through TEM, Raman spectroscopy (LabRaM-HR-800) and Fourier-transform infrared spectroscopy (170SX). The results are shown in Figs. S1 to S3.

### ^99m^Tc labelling of oMWCNTs and determination of labelling yields

The ^99m^Tc labelling of oMWCNT and determination of labelling yields were performed (^99m^TcO_4_^−^ was purchased from the China Institute of Atomic Energy, Beijing, China; 5 mCi) according to the method of Li *et al.*^55^. The unfilled electron orbits of Tc(V) were filled immediately by electrons donated by four hydroxy groups from oMWCNTs; thus, a stable chelate complex of ^99m^Tc-oMWCNTs was formed. oMWCNTs were dissolved in deionised water with an ultrasonic device for 5 min. Ascorbic acid, stannous chloride and ^99m^TcO_4_^−^ were then added into the suspension. This mixture was stirred at 90°C for 20 min. After centrifugation, the supernatant was decanted, and the remaining solid was identified to be ^99m^Tc-oMWCNT. The radiolabelling yields and stability of ^99m^Tc-oMWCNTs were measured by paper chromatography using Whatman 1 paper strips (Maidstone, Kent, UK) (Fig. 1). Considering the short lifetime of ^99m^Tc (6.02 h), we measured the radio counts in 1 d for accurate determination of the tag. Otherwise, the tag (^99m^Tc) would decay in 10 half times.

### Tissue biodistribution

Kunming mice (female:male = 1:1) initially weighing 15 g to 18 g were provided by the Laboratory Centre for Medical Science, Lanzhou University, Gansu, China. All animals were housed in individual cages in a temperature-controlled (21°C to 22°C) and light-controlled (turned on from 08:00 h to 20:00 h) environment and were fed food and tap water *ad libitum*. All animal protocols were in accordance with the European Communities Council Directive of November 24, 1986 (86/609/EEC) and approved by Institutional Animal Care and Use Committees of Gansu Province Medical Animal Center and Lanzhou University Animal Committees Guideline (China). When a mouse was up to sexual maturity (approximately 30 g), the cage (female:male = 1:1) was closed at approximately 10 p.m. The pessary was then then detected to determine pregnancy at approximately 7 a.m. the next day. The pregnant mice were organised into five groups (five mice/group) on the gestational age of approximately 17 d. The body weights of the mice were determined before they were injected intravenously with 20 mg/kg.bw of ^99m^Tc-oMWCNTs solution (pH = 7.26, *C*_NaCl_ = 0.9%, injection volume of 0.2 mL to 0.3 mL). The mice were killed at 1, 2, 6, 16 and 24 h after injection. Tissues from the heart, lungs, liver, spleen, kidney, stomach and foetus were immediately dissected. The maternal blood and the heart, lung and liver of the foetus were also collected. Each tissue or organ was wrapped in foil, weighed and counted for ^99m^Tc. Data were corrected for physical decay of radioactivity. The distribution in the tissues was presented in percent injected dose per gram of wet tissue (%ID/g), which could be calculated by the percent injected dose (tissue activity/total activity dose) per gram of the wet tissue^56^. The representative tissues obtained from the pregnant mice after exposure to oMWCNTs (20 mg/kg.bw) were digested using perchloric acid and hydrogen peroxide (volume, 1:2)^57^ and then subjected to TEM and Raman spectroscopy^58^,^59^ to further validate the reliability of ^99m^Tc labelling of oMWCNTs.

### Statistics of abortion rate and weight change before and after production

The first-time pregnant mice were injected with 0.2 mL to 0.3 mL of oMWCNTs (20 mg/kg.bw) at the gestational age of 7 d until abortion or parturition to serve as the experimental group (10 mice). Saline solution (*C*_NaCl_ = 0.9%, 0.2 mL) was administered to the control group (10 mice). The daily maternal body changes were recorded, and the status of the pregnant mice was observed. Genital tract bleeding during pregnancy marked the occurrence of abortion. A 1 mL blood sample was collected by anaesthetising the mice after abortion or parturition. The blood samples were kept in the refrigerator at 4°C for 24 h and then centrifuged at 3000 rpm/min to obtain the serum. The serum contents of progesterone and oestradiol were measured. It provided a similar dispose to the second-time pregnant and fourth-time pregnant mice as that of the first-time pregnant mice groups. The effects of oMWCNTs on the pregnant mice with different pregnancy times were then studied.

### Effects of oMWCNTs on progesterone and oestradiol

Progesterone and oestradiol ELISA kits were purchased from Shanghai Heng Hitter Trade Co., Ltd. and then kept in the refrigerator 4°C. Approximately 0.2 mL to 0.3 mL of oMWCNTs (20 mg/kg.bw) was intravenously injected to the experimental pregnant mice at the gestational ages of 4, 11 and 15 d every morning. Approximately 0.2 mL of saline solution was injected to the mice from the control groups (five to six mice/group). All mice were then killed at the gestational ages of 7, 14 and 18 d. Approximately 1 mL of maternal blood was collected. The blood samples were kept in the refrigerator at 4°C for 24 h and then centrifuged at 3000 rpm to obtain the serum. Afterward, the author studied the effect of oMWCNTs on the different gestational ages of pregnant mice by measuring the progesterone and oestradiol contents in maternal serum. The experimental groups (five to six mice/group) were exposed to 4, 20 and 30 mg/kg.bw of oMWCNTs (injection volume of 0.2 mL to 0.3 mL) at the gestational age of 11 d. Approximately 0.2 mL of saline solution was administered to the control groups, and approximately 1 mL of blood samples was obtained from all mice. The blood samples were processed as previously mentioned to investigate the dose of oMWCNTs that affected the pregnant mice. One group of pregnant mice (five mice) was exposed to 20 mg/kg.bw equivalent oMWCNTs at the gestational age of 13 d and to saline solution on the following days. Another group was injected with approximately 0.2 mL of oMWCNTs (4 mg/kg.bw) for five consecutive days (a total of 20 mg/kg.bw). The same volume of saline solution was administered to the mice from the control groups. Approximately 1 mL of blood sample was collected at the gestational age of 18 d and processes as previously described to determine the effects of the exposure time of oMWCNTs on pregnant mice.

### Detection of damage factor on pregnant mice with different pregnancy times

VEGF and ROS ELISA kits were purchased from Shanghai Heng Hitter Trade Co., Ltd. and then kept in the refrigerator at 4°C. Approximately 0.2 mL of oMWCNTs (20 mg/kg.bw) was injected intravenously to the experimental pregnant mice at the gestational ages of 9 d to 11 d every morning for 3 d (five to six mice). Approximately 0.2 mL of saline solution was administered to the mice from the control groups. All mice were then killed. Approximately 1 mL of maternal blood with anti-caking agent was collected to obtain the plasma. The plasma contents of ROS and VEGF were then measured. At the same time, the placenta, liver, lung, spleen and foetus were harvested immediately. These tissues were fixed in 10% buffered formalin and processed for routine histology with haematoxylin and eosin staining by the Centre for Medical Science, Lanzhou University (Lanzhou, China). Microscopic observation of tissues was performed with an Olympus Microphot-CX41 microscope coupled with a digital camera. The placental homogenate was made from part of the placenta tissues to investigate the ROS and VEGF contents.

### Statistical analysis

Data are reported as mean values +*SD* of multiple determinations. Statistical significance in the difference was evaluated by analysis of variance (ANOVA) or Kruskal-Wails-test method after One-sample Kplmogorov-Smirnov Npar-test and Homogeneity of Variances test, and all statistical calculations were carried out using *SPSS* software.

## Author Contributions

Q.W. and L.Z. wrote the main paper; Q.W., B.J., W.J. and L.P. carried out the experiments; Q.W. prepared all the figures and tables; Z.X., W.J. and W.W. revised the language of the paper; and all authors reviewed the paper.

## Supplementary Material

Supplementary Information Supplementary Information

## Figures and Tables

**Table 1:** The statistics of abortion rates after exposure to oMWCNTs (20 mg/kg.bw n = 10)

Group	Ectroma (colpo-bleeding)	Normal parturition	Average weight change before and after production/g	Total abortion rate
**First-oMWCNTs**	7	3	8.57 ± 8.95*	70%
**First-control**	1	9	20.73 ± 9.00	10%
**Second-oMWCNTs**	4	6	13.99 ± 9.44	40%
**Second-control**	0	10	15.83 ± 4.24	0
**Fourth-oMWCNTs**	5	5	11.18 ± 5.82	50%
**Fourth-control**	3	7	12.72 ± 5.78	30%

*p < 0.05 compared with the control groups by ANOVA, all data represent means + s.e.m.
