# Supplementary material for: Damaging Effects of Multi-walled Carbon Nanotubes on Pregnant Mice with Different Pregnancy Times
Source: Sci Rep. 2014 Mar 12;4:4352. doi: 10.1038/srep04352 (PMC3950636; doi:10.1038/srep04352)
Supplement: Supplementary Information [file srep04352-s1.pdf]

Supplementary Information

Damaging Effects of Multi-walled Carbon Nanotubes on Pregnant Mice with Different Pregnancy Times

Wei Qi[1], Juanjuan Bi[1], Xiaoyong Zhang[2], Jing Wang[1], Jianjun Wang[1], Peng Liu[1], Zhan Li[3*], Wangsuo Wu[1,4*]

[1] Radiochemical Laboratory, Lanzhou University, Lanzhou, Gansu, China, 730000

[2] Department of Chemistry and Key Laboratory of Bioorganic Phosphorus Chemistry and Chemical Biology (Ministry of Education), Tsinghua University, Beijing, China, 100084

[3] Key Laboratory of Chemistry of Northwestern Plant Resources, Key Laboratory for Natural Medicine of Gansu Province, Institute of Chemical Physics, Chinese Academy of Sciences, Lanzhou, Gansu, China, 730000

[4] State Key Laboratory of Applied Organic Chemistry, Lanzhou University, Lanzhou, Gansu, China, 730000

[*]Corresponding authors: Zhan Li, Email: lizhancg@gmail.com ; Wangsuo Wu, Email: wuws@lzu.edu.cn.

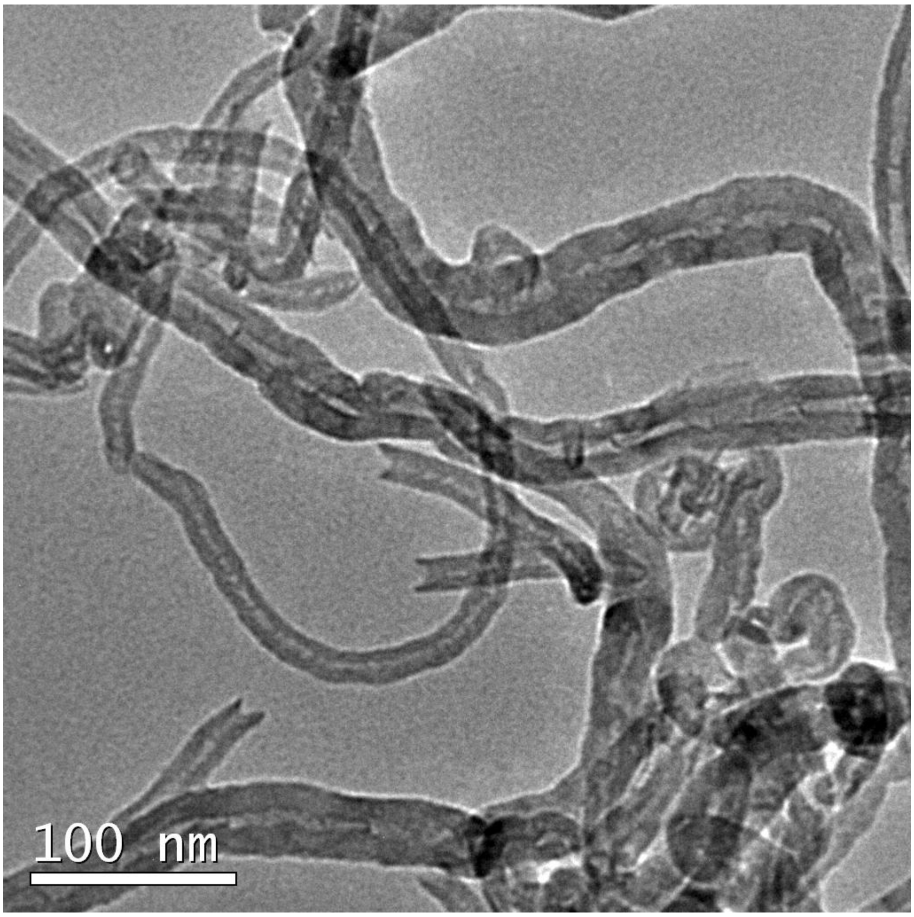

Fig.S1 the TEM of oMWCNTs.

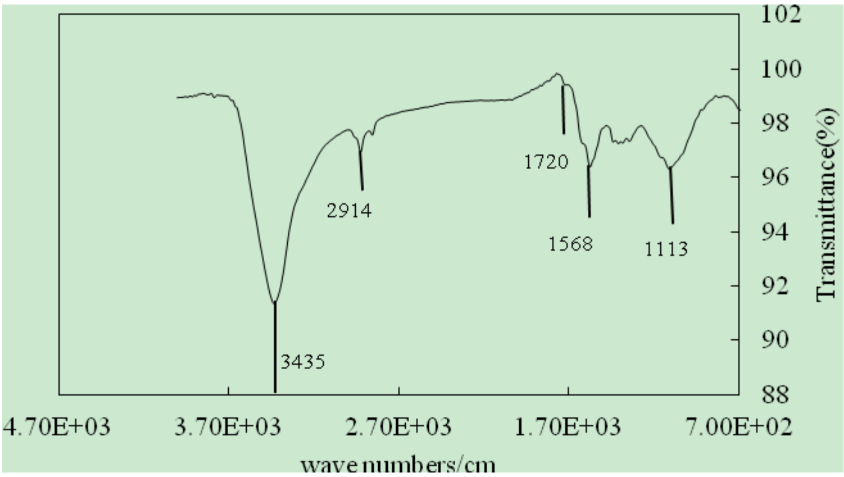

Fig.S2 the FT-IRs of oMWCNTs. The absorption peak of 3400/cm was OH-, and 1720-1730/cm was COOH-.

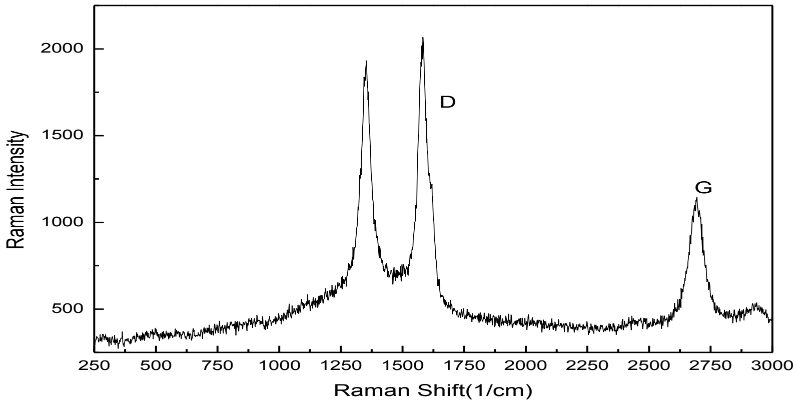

Fig.S3 the Raman spectroscopy of oMWCNTs. the D and G band of MWCNT still existed after oxidation, indicating that carbon nanotube structure was not changed.

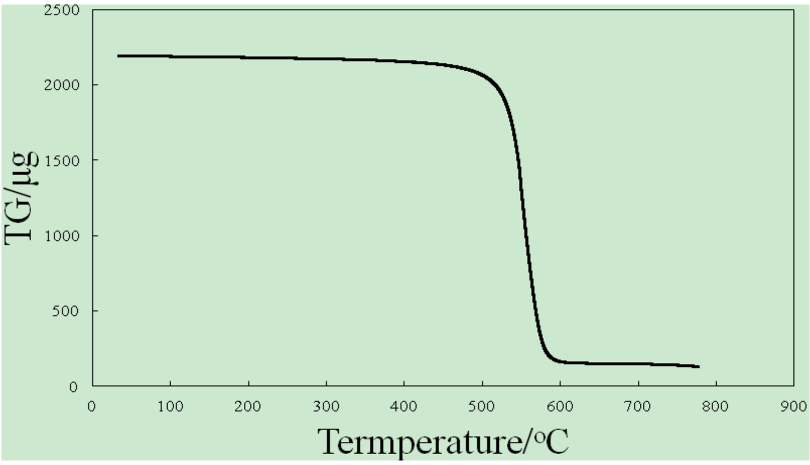

Fig.S4 the TGA of oMWCNTs in air.

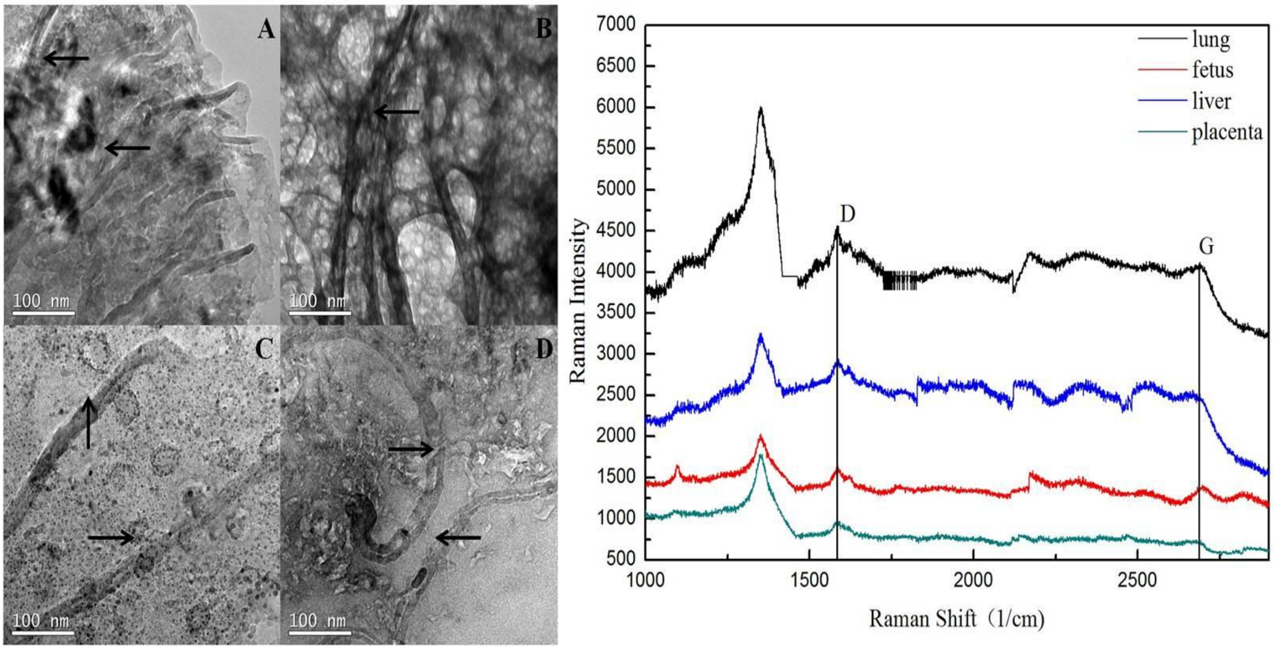

Fig.S5 the observed results of oMWCNTs in TEM and Raman spectrum of tissues digestive solution. A, lung; **B**, liver; **C**, foetus; **D**, placenta.

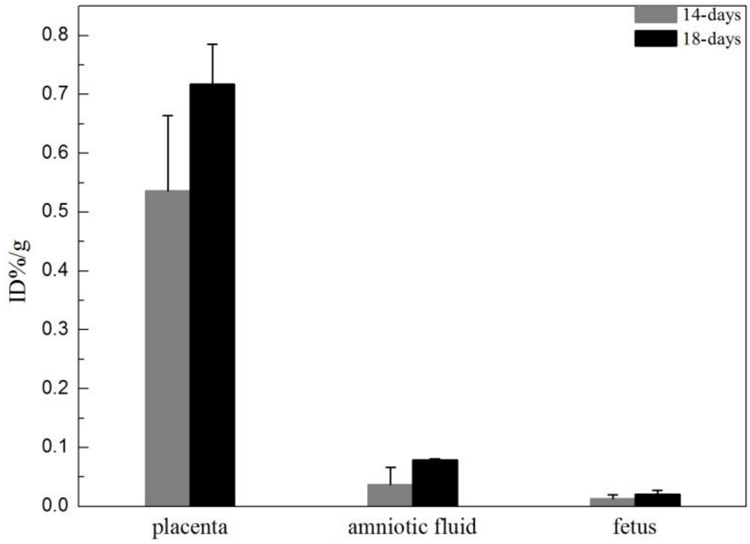

Fig.S6 the distribution content of placenta, amniotic fluid, and foetus in 12h after exposure ^{99m}Tc-oMWCNTs (20mg/kg.bw) to pregnant mice at the gestational age of 14days and 18days.(n=5, all data represent means +sd.)

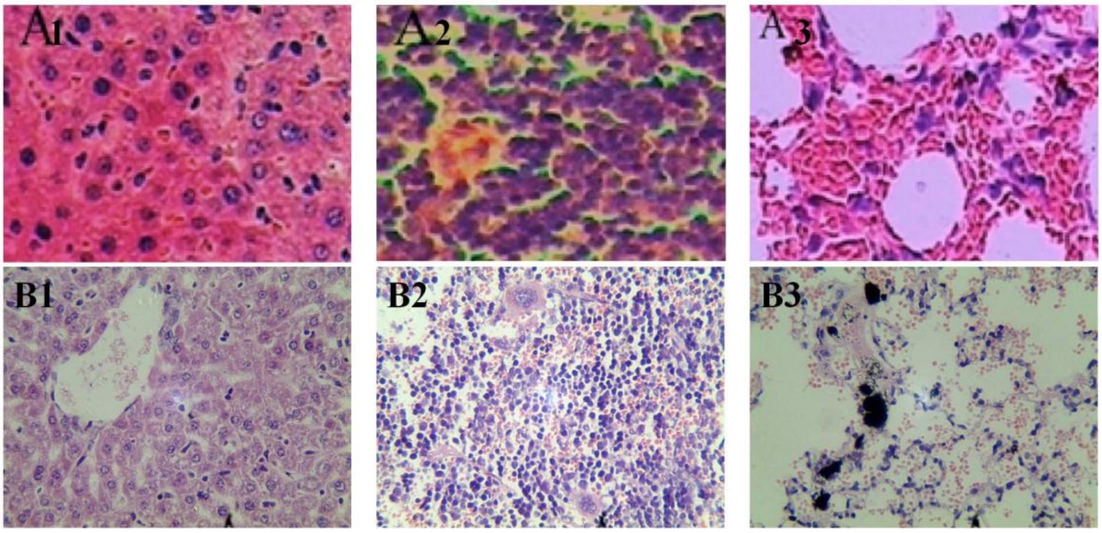

Fig.S7 the histology of liver, spleen and lung of maternal body. A1-A3(×40) is for liver, spleen and lung of control groups, **B1-B3**(×10) is for liver, spleen and lung of exposure groups respectively; the figures showed that oMWCNTs was captured obviously by lung, and edema or hyperemia occurred in liver, spleen and lung of exposure groups.

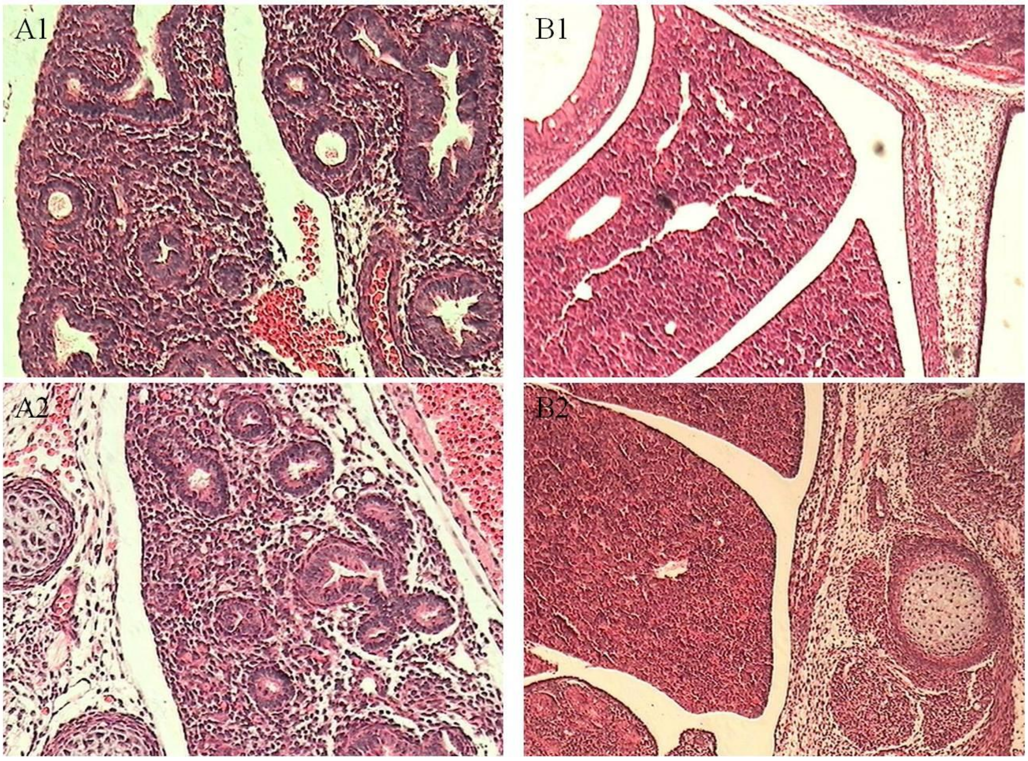

Fig.S8 the histology of lung (×10) and liver of foetus (×4) for first-pregnant mice. A1, **B1** is for lung and liver of normal groups, respectively; **A2**, **B2** is for lung and liver of exposure groups, respectively.

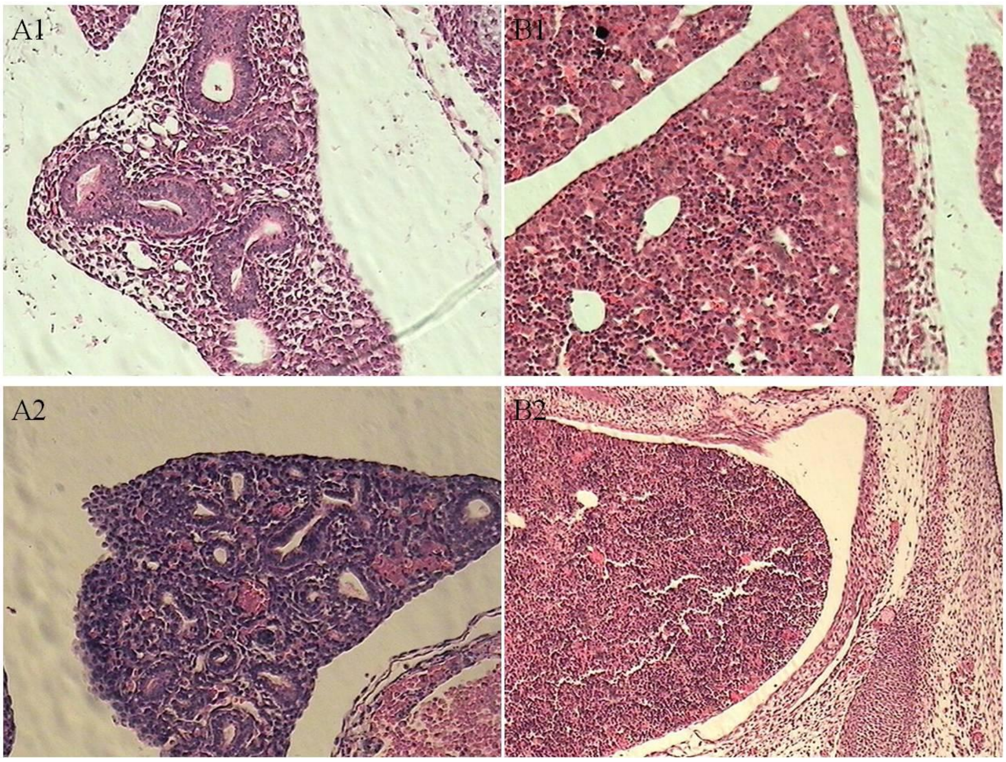

Fig.S9 the histology of lung (×10) and liver of foetus (×10) for second-pregnant mice. A1, **B1** is for lung and liver of normal groups, respectively; **A2**, **B2** is for lung and liver of exposure groups, respectively.

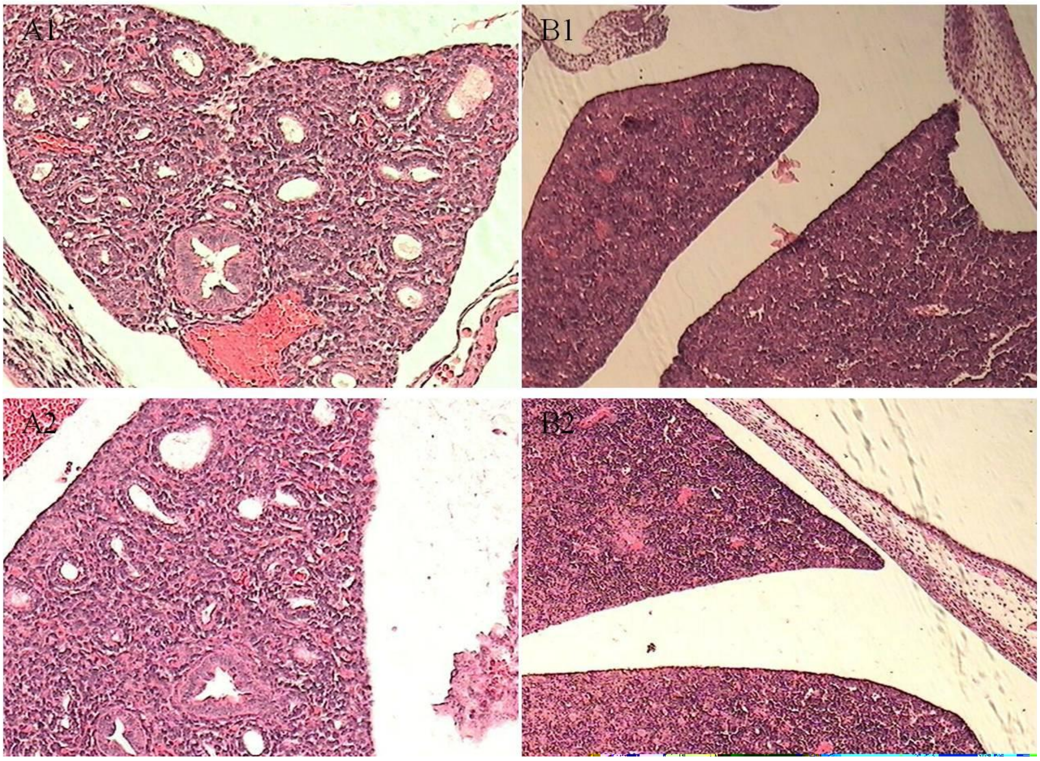

Fig.S10 the histology of lung (×10) and liver of foetus (×4) for fourth-pregnant mice. A1, **B1** is for lung and liver of normal groups, respectively; **A2**, **B2** is for lung and liver of exposure groups, respectively.
